# Plant-Based: A Perspective on Nutritional and Technological Issues. Are We Ready for “Precision Processing”?

**DOI:** 10.3389/fnut.2022.878926

**Published:** 2022-04-26

**Authors:** Roberto Menta, Ginevra Rosso, Federico Canzoneri

**Affiliations:** Soremartec Italia Srl, Ferrero Group, Alba, Italy

**Keywords:** plant-based, sustainability, nutrition, novel physical processing technologies, protein, precision processing

## Abstract

The rapid evolution of consumers' preference despite being still rooted in taste is rapidly combining with an exponential growth of environmental awareness. Both are forcing innovation into the food industry sector. Today, it is common in the scientific literature to find awareness of nutrition and sustainability, functionality and freshness, taste, and pollution; the most relevant and recognized trends are evolving with unprecedent speed toward a new paradigm. The perfect storm of fast-growing population, together with an exploding level of environmental pollution, is combining with the request for functional foods with more defined health properties and is strongly pushing the food sector to new defined innovation objectives to keep and develop the economic role of most loved brands around the world. The most debated conundrum is how to provide healthy food for all human beings, without further affecting our Mother Earth. Innovation in food raw materials as well as innovation in food processing seems to be the magic solution to provide *twice with half*, that is, to double the food production combined with declining resources. One of the fastest growing segments in the food industry is the plant-based segment. The status of the available options in food processing applied to plant-based food will be discussed, with a special focus on novel physical processing technologies and atomic force microscopy as possible complementary weapons in science-based definition of a sustainable nutrition approach. A call for a new paradigm such as “precision processing” should be adopted to drive the evolution of the whole food system.

## Introduction

Nutrition science is expanding its core competence from the simple profiling of nutrients to a more holistic approach that includes the sustainability of nutrition based on sustainable development goals (1). As the EAT Lancet committee firmly indicated, diet, health, and environmental sustainability are closely intertwined, with food being the lever to optimize human and environmental health (2). Following a plant-based diet, according to what has been demonstrated in the ever-growing literature on the subject, would seem to represent a winning approach for both people and the planet, and, driven by this concept, the growing demand of plant-based food was the primer of incredible number of scientific and related publication during the last 5 years, when 23,383 articles were published (3). In this context, protein takes on a critical role, both from nutritional and environmental perspectives. In fact, in view of the growth of the global population in the near future, which is expected to be 10 billion in 2050, it becomes of primary importance to ensure an adequate protein intake for all the world's population, in addition to meeting the global caloric needs. Considering the intense amount of land and resources required for animal proteins' production, it, once again, becomes necessary to shift the focus to alternative sources of protein, such as plant-based proteins, representing a promising solution to our nutritional needs due to their long history of crop use and cultivation, lower cost of production, and easy access in many parts of the world (4). Such proteins could be found in pulses (e.g., peas, beans, chickpeas, lentils, and lupines), oilseeds (soybeans, peanut, flaxseed, and rapeseed/canola), cereals (wheat, corn, rice, oats, barley, and sorghum), and pseudocereals (quinoa, amaranth, chia, and buckwheat) (5). However, plant proteins present some challenges, given the lower protein quality and technological functionality; therefore, it is of primary importance in the production of this new generation of plant-based foods to have a thorough understanding of the characteristics of the plant-derived ingredients that comprise it to identify desirable physicochemical and sensory attributes in the finished product (6). Research in recent years is consolidating a certain know-how aimed at improving the technological profile of vegetable proteins, starting from their manipulation in the production phase, such as during extraction, fractionation, and modification that can considerably enhance their functionality (7–9). The literature is, indeed, showing how modifying the structural arrangements of plant proteins toward forms that give physicochemical and functional properties comparable to those provided by animal proteins is the core challenge in this research field (4). The increasing demand for better performing proteins has led to the implementation of various thermal and non-thermal treatments to modify plant proteins. In particular, novel physical processing technologies (NPPT) claimed to be *emerging high-potential treatments for tomorrow* are needed in this context and will be further explored in the next chapter (5, 10).

## Classification of Thermal and Non-Thermal Technologies and Their Concrete Applications: Focus on NPPT

Global food markets demand for plant-based ingredients and food that are, first of all, safe, preferably with a natural halo, and with good nutritional value. The sustainability of processes as well seems to be a parameter recognized and requested by consumers and the market for this type of product (11). Conventional heat-dependent technologies, such as pasteurization and sterilization, however, can have a strong impact on sensory and nutritional characteristics that adversely affect the overall quality of the food product (11); therefore, alternative techniques have gained much attention, with the development of so called “novel” and “emerging” technologies.

Over the years, two main options are subject of intensive research and are emerging in food systems: non-thermal technologies and novel thermal processing technologies (11). Non-thermal technologies, such as high pressure, pulsed electric field (PEF), carbon dioxide processing, and membrane processing, are recognized as value-added processes and sustainable alternatives to conventional treatments, mainly due to the reduction of energy and water consumption (12). These non-thermal techniques are based on the inactivation of microorganisms and spoilage enzymes by means of physical hurdles, such as pressure, electromagnetic fields, and sound waves. They can result in sterilization at room temperature or lower than thermal analogs and are more and more adopted because of the efficient inhibitory effects on microbes, with the promise that taste and texture features are consistent with the chemical properties of ingredients. On the other side, we have the novel thermal technologies that mainly use energy generated by microwave and radio frequency, such as ohmic heating, microwave heating, dielectric heating, or radio frequency heating (13).

As mentioned at the beginning, in addition to food safety aspects also considering the sustainability of the process used, it is of fundamental importance in the context of plant-based foods not to forget the challenges imposed by these new ingredients both from nutritional, sensorial, and technological points of view. Plant-based proteins have, in fact, limits in their applications because of their poor techno-functionality, digestibility, bioactivities, and presence of anti-nutritional compounds with certain off taste (10). Conventional heat treatments, such as drying, extrusion, roasting, boiling, and steaming, are among the most common methods applied for physically modifying the structure of proteins in order to enhance their functionality. However, these techniques, given the heavy conditions intrinsic in the process, could induce denaturation and damages on the secondary and tertiary structures, causing alteration in the nutritional and sensory profile of proteins (4, 14).

**Figure 1:**
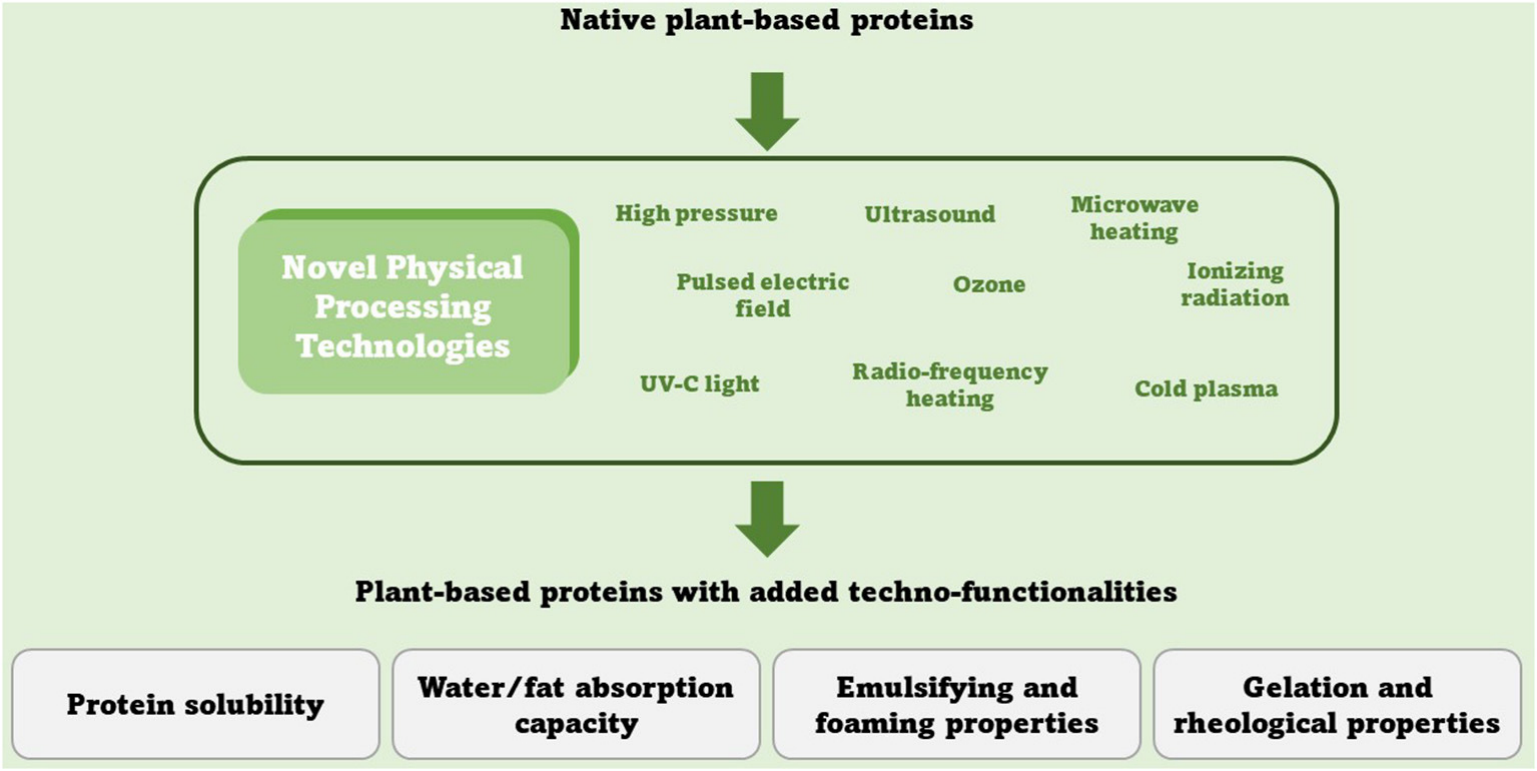
A summary of NPPT able to impact plant-based protein functionalities.

In this context, NPPTs, such as high pressure, ultrasound, microwave, radiofrequency, ozone, ultraviolet-C, cold plasma, ionizing radiation, and the pulsed electric field, are emerging as promising new technologies able to overcome the limit expressed above – see Figure 1. These NPPTs are able to modify the protein structure by disrupting various interactions among protein molecules, leading to techno-functionality changes (10). When we are talking about techno-functionality related to plant-based proteins, four are the main aspects we should consider: water/fat absorption capacities, protein solubility, emulsifying and foaming properties, and gelation and rheological properties (5, 10).

To explore one of the most studied techniques in this context, high pressure processing (HPP) has gained much attention in the food industry with the purpose of inactivate spoilage microorganisms and dangerous pathogens (5). In HPP, the heat is substituted by pressure (ranging from 100 to 800 MPa) (5), resulting in cold pasteurization, therefore avoiding the damage caused by high temperatures. Furthermore, over years, this technique has been shown to positively affect protein structure and related functionalities, such as the capacity to stabilize emulsions and foams, and to form aggregates and gels, mainly impacting the secondary, tertiary, and quaternary structures of proteins, considering the HPP effect on noncovalent bonds (15). The level of improvement of HPP-induced modifications and its commercial applications depend on several factors, such as pH, the pressure level, treatment time and temperature, as well as protein type (15). In addition, it is worth mentioning that the application of HPP has been shown to be effective as a pre-treatment of legumes followed by enzymatic hydrolysis in increasing protein digestibility (16).

Another technique that should be mentioned is ultrasound, which is a promising sustainable novel technology for plant protein treatment, mostly for its ability to enhance green chemistry by using nontoxic, clean, and green solvents (17). Furthermore, in comparison with other eco-innovative technologies, it requires lower investment and shorter times. As with HPP, the right combination of processing conditions has been shown to increase solubility, oil absorption, water-holding capacity, emulsifying, and gelling properties (13), and also proves to be an efficient method for protein extraction (14).

These are some examples that highlight the potential of these innovative techniques to address the need for greater use of plant proteins in the food industry, increasing their functionality and potential. In fact, we are witnessing a paradigm shift in which food safety, a fundamental requirement of the food industry, is being joined by new concepts, such as sustainability and functionality of the process and ingredients. The change in perspective forces us to ask ourselves how traditional processes can adapt to these new requirements, and we may find the answer in *precision processing*. This means the search for processes capable of adapting to the available raw materials, enhancing their natural characteristics but, at the same time, strengthening their functions in order to be suitable for tomorrow's food system, which will have to rely on alternative sources of nutrients, especially proteins, mostly of vegetable origin.

## Precision Food Characterization

The momentum for change in food processing is fostered by a general concern about continuing with thermal technology. Growing evidence suggests that non-thermal technology is providing substantial benefits in energy needs, while combination of thermal and non-thermal technology is in place (18). Moreover, the merging of public health nutrition and sustainability is the major challenge in the XXI century, with the protein transition diet (less animal protein, more plant protein) being central to our species' chance of survival (19) as well as the codification of a global diet (2). Based on this perspective, it will be necessary to initiate in-depth research aimed at precision characterization of foods. To deal with this ambitious aspect, a first attempt in precisely characterized foods could be the application of atomic force microscopy (AFM), a very high-resolution type of scanning probe microscopy, which has been extensively reviewed in a recent special issue (20). There are several applications of this technique in food science as reported in Table 1.

**Table 1:** Applications of atomic force microscopy (AFM) in food science research.

**AFM applications**	**References**
Asses nanomechanical properties of food materials.	(20)
**Proteins**	
Topography characterization of protein.	(21)
Processing and preservation effects on food proteins.	(21)
Interaction research between food proteins and other substances.	(21)
**Food packaging**	
Characterization of different chemicals and physical properties.	(22)
Bacterial adhesion on food packaging surfaces.	(22)
**Food safety**	
Food toxins detection.	(23)
Quantify the dimension alterations and surface features of food borne pathogens and spoilage microbes to elucidate bactericidal mechanisms and cellular responses under adverse environments.	(24)
Analyses of the adhesion capacity of food borne pathogens, contribute to the biocontrol of biofilm in food-processing surfaces, the elimination of disease transmission, and the prolongation of product shelf life.	(24)
**Polysaccharides**	
Morphology characterization of pectin, xanthan, carrageenan, β-glucan, hemicellulose, starch and others.	(25)
Investigation of structure-function properties of polysaccharides in various conditions and complex systems.	(25)
Opportunities to control and improve the quality of food product during processing and preservation.	(25)

## Discussion

As briefly described, we are facing a new era in which food(s) will be more and more integral to our way of life, not based only, as in the past, on tradition or on consolidated features but also on addressing broader the scientific horizon of human beings. What we expect from the food of future is to continue to enjoy tasteful food, with the intrinsic property of safety at the maximum level, but more respectful of the environment and with an egalitarian principle as founding elements. We, therefore, need to develop the food of the future according to “precision processing,” using innovative processes able to adapt to the raw materials available, enhancing their quality and functionality. That is why we suggest a re-examination of raw materials from vegetable sources based on an intrinsic assessment of environmental costs, processing costs without forgetting the growing demand for more functional foods. Previous experience has documented that, without focusing on taste, it is difficult to gain consumer support without the vast majority of consumers' final choice being focused on conscious evaluation and preferred consumed items. Only if we face the problem with holistic and non-biased eyes can we look for the definition of a set of principles that will allow the scientists to rank possible suitable options for a healthy, egalitarian diet that is available for all and will not further affect our Mother Earth. It is also clear that, now, even the most innovative processes are far from being perfect, but, in any case, we know that *business as usual* is no longer a choice or even an option. Therefore, any kind of prejudices should be avoided; new unexplored ideas must enter in the scientific arena. Our perspective cannot be exhaustive of such a very complex matter, and we decided that the simple way of listing the findings showing the distance between what is possible and what is not is the only way to start together a new era and face new paradigms. We know that new technologies are having huge potential; they impact the structure, the organization, the facility cost, and the day-to-day operations, but, moreover, they can really promote a better nutrition for all. We must acknowledge the time in which we can try to solve the problems of food supply for humanity is getting shorter and shorter; the approach that is proposed as *precision processing* is at the end, only a way to call for a common effort and to focus on realistic solution.

## Data Availability Statement

The original contributions presented in the study are included in the article/supplementary material, further inquiries can be directed to the corresponding author.

## Author Contributions

Conceptualization and writing—original draft preparation was contributed by RM. Writing—review and editing and visualization were contributed by GR and FC. All authors contributed to the article and approved the submitted version.

## Conflict of Interest

RM, GR, and FC are employed by Soremartec Italia Srl, Alba (CN, Italy).

## Publisher's Note

All claims expressed in this article are solely those of the authors and do not necessarily represent those of their affiliated organizations, or those of the publisher, the editors and the reviewers. Any product that may be evaluated in this article, or claim that may be made by its manufacturer, is not guaranteed or endorsed by the publisher.
